# Unveiling Embryonic Development of the Threatened Neotropical Fish *Prochilodus vimboides* (Characiformes: Prochilodontidae)

**DOI:** 10.3390/ani16050852

**Published:** 2026-03-09

**Authors:** Renato Massaaki Honji, Amanda da Silveira Guerreiro, Bruno Cavalheiro Araújo, Danilo Caneppele, Sergio Ricardo Batlouni, Renata Guimarães Moreira

**Affiliations:** 1 Laboratório de Aquicultura e Ecofisiologia Marinha (LAQUEFIM), Departamento de Fisiologia, Instituto de Biociências, Universidade de São Paulo (IB/USP), Rua do Matão, Travessa 14, No. 321, São Paulo 05508-090, SP, Brazil; 2 Laboratório de Metabolismo e Reprodução de Organismos Aquáticos (LAMEROA), Departamento de Fisiologia, Instituto de Biociências, Universidade de São Paulo (IB/USP), Rua do Matão, Travessa 14, No. 321, São Paulo 05508-090, SP, Brazil; 3 Laboratório de Reprodução de Peixes, Centro de Aquicultura, Universidade Estadual Paulista “Júlio de Mesquita Filho” (CAUNESP), Via de Acesso Prof. Paulo Donato Castellane, s/n, Jaboticabal 14884-900, SP, Brazil; 4 Laboratório de Fisiologia e Nutrição de Organismos Aquáticos (LAFINUTRI), Núcleo Integrado de Biotecnologia, Universidade de Mogi das Cruzes (UMC), Av. Dr. Cândido Xavier de Almeida Souza, No. 200, Mogi das Cruzes 08780-911, SP, Brazil; 5 HMZ Aquicultura & Conservação, Rua Telmo Arnaut de Carvalho, 262, Paraibuna 12260-000, SP, Brazil

**Keywords:** early development, eggs, larvae, ontogeny, grumatã, captive breeding

## Abstract

Understanding embryonic development is fundamental to improving captive breeding protocols and supporting conservation strategies for threatened fish species. *Prochilodus vimboides* is a Neotropical freshwater fish for which detailed information on early ontogeny remains scarce. This study aimed to characterize the embryonic and early larval development of *P. vimboides* under captive conditions. Broodstock were hormonally induced to reproduce, and extrusion occurred between 209 and 230 degree-hours after induction at 21.49 ± 0.15 °C. Embryonic development was monitored at regular intervals after fertilization (AF) using freshly collected eggs examined under a stereomicroscope. The principal developmental stages were identified, namely zygote, cleavage, including morula and blastula, gastrula, organogenesis, and hatching. Fertilized oocytes exhibited marked hydration and formation of a large perivitelline space at 15 min AF. More than 50% of embryos reached the two-blastomere stage by 20 min AF, and cleavage continued until 2 h 14 min AF. The gastrula stage was observed at 3 h 23 min AF, blastopore closure occurred at 11 h 47 min AF, and organogenesis began at 12 h 55 min AF. Complete hatching occurred at 22 h 04 min AF, and larvae subsequently initiated yolk sac absorption without cannibalistic behavior. These findings provide a species-specific developmental framework that supports captive production and conservation efforts for *P. vimboides* in the Paraíba do Sul River Basin.

## Simple Summary

*Prochilodus vimboides* is a Neotropical freshwater fish native to the Paraíba do Sul and Paraibuna Rivers and is currently classified as endangered. Knowledge of embryonic and early larval development is essential to improve captive breeding programs and support conservation efforts for this species. In this study, broodstock were hormonally induced to reproduce under controlled laboratory conditions, and offspring development was monitored from fertilization to hatching. Shortly after fertilization, fertilized eggs increased in size and underwent rapid cleavage. As development progressed, major embryonic structures such as the head, tail, eyes, musculature, and yolk sac gradually differentiated. Hatching occurred at approximately 22 h after fertilization, followed by progressive yolk sac absorption during the larval phase. In summary, the present work provides a detailed characterization of the early development of *P. vimboides*, contributing to broader knowledge of this threatened Neotropical species.

## 1. Introduction

According to Eschmeyer’s Catalog of Fishes [1], the family Prochilodontidae (Teleostei: Ostariophysi: Characiformes) comprises three valid genera, *Prochilodus*, *Ichthyoelephas* and *Semaprochilodus*, and currently includes 21 valid species distributed across the major river basins of South America [2]. Within this family, the genus *Prochilodus* represents one of the most important Neotropical groups of rheophilic teleost fishes and constitutes a major component of both commercial and subsistence freshwater fisheries in South America [2,3]. All 13 species belonging to this genus are characterized by highly modified lips, teeth, and jaws adapted for the consumption of detritus and periphyton [3]. Among them, *P. vimboides* represents the focus species of this study.

*Prochilodus vimboides*, commonly known as grumatã or curimbatá, is a rheophilic, detritivorous, medium-sized teleost fish endemic to Brazil. Records of occurrence extend from the Jucuruçu River in southern Bahia State to the Paraíba do Sul River, including the Doce and Paraibuna Rivers in the states of Rio de Janeiro, Minas Gerais and São Paulo [2]. Likewise, this species is distributed in the Uruguay River and in the headwaters of tributaries of both the upper Paraná River and the São Francisco River [2]. Despite this wide geographic distribution, *P. vimboides* is classified as “vulnerable” by both the Biodiversity Extinction Risk Assessment System (SALVE) of the *Instituto Chico Mendes de Conservação da Biodiversidade* (ICMBio, Brazil) [4] and the International Union for Conservation of Nature (IUCN) [5]. The principal threats to *P. vimboides* include dam construction for hydroelectric power generation or water supply, riparian habitat degradation, water pollution associated with freshwater eutrophication and residential and industrial effluents, and overfishing for sport and commercial purposes. These pressures have markedly reduced natural stocks of *P. vimboides* in major South America river basins, particularly in the Paraíba do Sul River Basin [6].

Since 2009, the *Centro Nacional de Pesquisa e Conservação da Biodiversidade Aquática Continental* (CEPTA), linked to ICMBio, has coordinated the National Action Plan (PAN) for the Paraíba do Sul River Basin, which aims to preserve the principal threatened species of this basin, including the maintenance of broodstock and successful larval rearing [7]. However, when wild broodstock are transferred to captivity, many teleost fishes exhibit reproductive dysfunctions [8], including *P. vimboides*, which limits fingerling production of native species in Brazil [6,8]. In this context, captive propagation has been proposed as a strategy to reduce anthropogenic pressure on natural populations and to support conservation actions [9], although its success depends on a robust understanding of species-specific biology, particularly reproductive physiology and early development [10].

The five main endangered fish species listed in PAN Paraíba do Sul [6,7] exhibit reproductive dysfunction under captive conditions, particularly those classified as type II according to Zohar & Mylonas [8]. This dysfunction is characterized by the absence of final oocyte maturation (FOM) and ovulation, representing a major bottleneck for reproductive success. Despite the relevance of these processes, information on the physiological mechanisms underlying FOM and ovulation in species included in PAN Paraíba do Sul remains scarce. Reproductive studies are available for *Steindachneridion parahybae* [6,7,11], whereas comparable data for *P. vimboides* remain limited or incipient.

In this context, a fundamental step in the physiological study of any fish species is the characterization of embryonic and larval stages, since early ontogeny represents a critical developmental phase [10]. This information is particularly important for threatened species, because such studies generate knowledge that supports the improvement and optimization of captive rearing protocols, provides essential data for the development of aquaculture biotechnology [11], and establishes baseline developmental models for detecting alteration caused by toxic substances in aquatic organisms [12]. Therefore, studies on the early development of *P. vimboides* are especially relevant within the Paraíba do Sul River Basin, particularly in Paraíba do Sul and Paraibuna Rivers. These rivers receive substantial residential and industrial effluent discharge and contain hydroelectric dams that act as physical barriers to the reproductive migration of rheophilic species [6,7]. Consequently, *P. vimboides* exhibits reproductive dysfunction under captive conditions, and the underlying causes of these impairments remain unknown [6].

Previous studies on *P. vimboides* have described early development and allometric growth patterns of specimens derived from an ex situ bank of endangered species from the Imbé River Basin at Itaocara City, Rio de Janeiro State, Brazil (21°38′6.33″ S 42°1′59.25″ W), providing important baseline information for this species [13,14]. However, these studies were conducted on a different population within the Paraíba do Sul system and did not establish a detailed, temperature-specific embryological timeline. Considering that local environmental and physicochemical conditions may influence early developmental processes, additional population-specific data are required. In this context, the present study aims to characterize in detail the embryonic and early larval developmental stages of *P. vimboides* under captive conditions, using broodstock originating from the Paraíba do Sul and Paraibuna Rivers. Early ontogenetic development was systematically documented from oocyte activation to the initial larval period, generating a fine-scale, temperature-dependent embryological timeline. By addressing this knowledge gap, this study establishes a species-specific developmental framework that supports comparative developmental analyses within Prochilodontidae and informs conservation and captive reproduction strategies for this threatened species.

## 2. Materials and Methods

**Figure 1:**
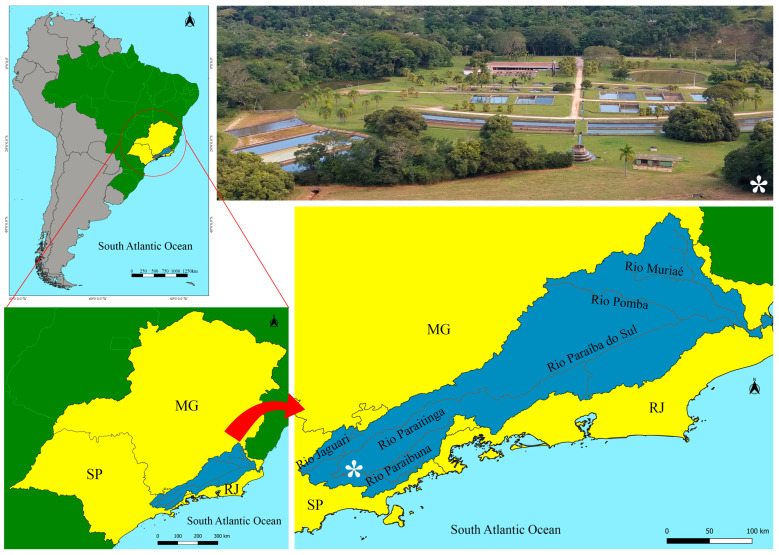
Geographic location of the Paraíba do Sul River Basin and its principal rivers and tributaries (blue), encompassing the states of São Paulo (SP), Rio de Janeiro (RJ), and Minas Gerais (MG) (yellow), Brazil (green). The asterisk indicates the location of the former *Unidade de Hidrobiologia e Aquicultura* of the *Companhia Energética de São Paulo* (CESP), in the municipality of Paraibuna, São Paulo State, situated along the Paraibuna River, where the present study was conducted. Source: *Sistema de Coordenadas Geográficas*, Datum SIRGAS [16], *Bases Cartográficas* [17], and United States Geological Survey [18]. Spatial analysis was performed in the QGIS environment with integration into SAGA GIS [19].

The present study was conducted at the former *Unidade de Hidrobiologia e Aquicultura* of the *Companhia Energética de São Paulo* (CESP) (23°24′45″ S; 45°36′40″ W) located in the municipality of Paraibuna, São Paulo State, Brazil (Figure 1). This study was associated with the Conservation Program of Endangered Species of the Paraíba do Sul Basin (PAN Paraíba do Sul), implemented by the former CESP/Paraibuna and by CEPTA/ICMBio [15]. The *P. vimboides* broodstock used for egg and larval sampling originated from the ex situ bank of endangered species of the PAN Paraíba do Sul Basin maintained by CESP/Paraibuna. At this facility, *P. vimboides* broodstock were maintained in 200 m^2^ earthen ponds at a stocking density of 1 fish m^3^ under natural photoperiod. Fish were fed a commercial extruded diet containing 32% crude protein at a weekly feeding rate corresponding to 5% of biomass, distributed in six feedings per week according to the CESP routine. The annual mean water temperature, dissolved oxygen concentration, pH, and conductivity monitored with an oximeter (Horiba-ModU10) (HORIBA Ltd., Kyoto, Japan), were 21.45 ± 0.29 °C, 6.78 ± 0.09 mg L^−1^, 6.70 ± 0.14, and 0.04 ± 0.01 mS cm^−1^, respectively.

### 2.1. Broodstock Selection, Hormonal Induction and Spawning

According to Honji et al. [6], the reproductive period of *P. vimboides* extends from late spring, November, to March, with a reproductive peak in December and January (spring/summer months in South America). During this reproductive peak, two independent spawning trials were conducted using a total of 12 females (479.33 ± 8.41 g) and 20 males (215.67 ± 5.36 g). Broodstock were selected based on morphological indicators of sexual maturity described for Prochilodontidae species. Females were selected based on external characteristics including a hyperemic genital pore and a swollen, soft abdomen. In addition, ovarian biopsy was performed to assess oocyte characteristics such as size, appearance, and diameter homogeneity [20]. Oocytes were collected by cannulation of the gonoduct through the gonadal papilla using fine polyethylene tubing (5 mm diameter) attached to a plastic syringe. Oocyte diameter and morphology were examined under a stereomicroscope (Leica S6D stereomicroscope connected Leica DFC295 camera) (Leica Microsystems, Wetzlar, Germany) using Leica LAS Interactive Measurements software v4.12. Males were selected based on the presence of freely flowing sperm released after gentle abdominal massage, exhibiting a white coloration indicative of sexual maturity and suitability for hormonal induction.

After selection in earthen ponds, broodstock were transferred to indoor tanks (1000 L), and artificial reproduction was performed according to the former CESP routine. Females received two intraperitoneal injections of carp pituitary extract (cPE) (Fish Braz^®^) at a 12 h interval. The first dose was 0.5 mg cPE kg^−1^ body mass, and the second dose was 5.0 mg cPE kg^−1^ body mass, both diluted in 0.9% sodium chloride solution with a final injection volume of 1.0 mL per dose. Males received a single injection at the time of the female second dose, consisting of 3 mg cPE kg^−1^ body mass diluted in 0.9% sodium chloride solution, also with a final volume of 1.0 mL. This protocol was adapted for *P. vimboides* from the method described by von Ihering and Azevedo [21].

During the entire artificial reproduction period, from the first hormonal administration to gamete stripping, females and males were maintained in separate tanks under identical environmental conditions. According to Weingartner and Zaniboni-Filho [22], the latency period between the second hormonal administration and spawning was calculated as degree-hours or Unit Thermal Accumulated (UTA), defined as the sum of hourly water temperature values from the second hormonal injection until spawning.

Subsequently, dry stripping of gametes from females was performed into plastic containers. Oocytes were weighed and placed in plastic bowls, sperm was added by the dry method, and the mixture was gently homogenized. Water was then added to promote hydration and fertilization, after which eggs were transferred to a 200 L fiberglass conical incubator. The estimated number of oocytes per female was calculated indirectly by weighing total egg mass and counting subsamples in triplicate [11,23]. After gamete stripping, broodstock were returned to their original earthen ponds, and survival rate was calculated as: Survival rate (%) = (number of broodstock surviving post-hormonal induction/total number of broodstock) × 100.

Eggs obtained from hormonally induced females within each spawning trial were pooled immediately after fertilization and transferred to independent fiberglass conical incubators, each incubator representing one spawning unit. Both independent spawning trials were included in all analyses, and data are presented as the mean values obtained from these two trials. Eight hours after incubation, corresponding to the final gastrula stage and blastopore closure, fertilization rate was determined using the formula F = (number of fertilized eggs × 100)/total number of eggs [24,25]. For fertilization assessment, five random subsamples (*n* = 20–30 eggs per subsample) were collected from each incubator to ensure representative sampling. Fertilized eggs were distinguished from unfertilized eggs based on overall morphology and coloration, as unfertilized eggs appeared opaque. After evaluation, sampled eggs were returned to their respective incubators. Embryonic development proceeded within each incubator separately until hatching and yolk sac absorption, which occurred approximately seven days post-fertilization. After this period, larvae were transferred to outdoor earthen ponds of 200 m^2^ previously prepared and enriched with natural food, while also receiving daily powdered commercial feed. This larviculture protocol corresponds to the routine procedure adopted by the former CESP fish farm.

### 2.2. Characteristics of the Freshly Spawned Egg and Larval Analysis

The key morphological features of each embryonic stage were described in detail. Oocytes, eggs, embryos, and larvae collected at each sampling interval were measured using a Leica S6D stereomicroscope connected to a Leica DFC295 camera and analyzed with Leica LAS Interactive Measurements software v4.12. Fresh egg samples were collected at 5 min intervals, and photographic documentation was performed continuously until hatching. Developmental stages were defined when more than 50% of specimens in a sample exhibited the same morphological characteristics. This assessment used five subsamples, each containing 20–30 eggs or larvae. After each observation, and photographic record, embryos and larvae together with the sampled water volume were returned to the original incubators. For morphometric analysis of embryonic and larval development of *P. vimboides* under captive reproduction, the following parameters were measured (*n* = 20–30 per stage): spatial arrangement during the egg phase, total larval length (L_t_, mm), yolk sac length (YL, mm), and yolk sac height (YH, mm). YL and YH were measured shortly after hatching, and yolk sac volume was estimated using the prolate spheroid formula: {V(mm^3^) = [π × L × (h^2^)]/6}, where L corresponds to YL and h corresponds to YH [26]. Total length and yolk sac volume measurements were obtained using Leica LAS Interactive Measurements software v4.12.

### 2.3. Water Quality Analysis and Additional Information

Water quality parameters, temperature (°C), dissolved oxygen (mg L^−1^), pH, and conductivity (mS cm^−1^), monitored with an oximeter (Horiba-ModU10), during artificial reproduction, embryonic incubation, and the first seven days of larval development are presented in Table 1. All values are expressed as mean ± standard error of the mean (M ± SEM).

Terminology used to describe embryonic and early larval stages varies among authors. In this study, we identified embryonic and larval developmental stages according to descriptions established for Prochilodontidae species (File S1), and following the ontogenetic frameworks proposed by Nakatani et al. [10] and Richards [27]. The following developmental periods were considered for freshly spawned eggs of *P. vimboides*:

(a) Egg stage, from fertilization to hatching, characterized by the presence of chorion, perivitelline space, and embryo surrounding a centrally located yolk sac;
(b) Larval stage with yolk sac, from hatching until the onset of exogenous feeding;
(c) Larval stage with exogenous feeding, beginning with initiation of exogenous feeding in *P. vimboides*.

Fish handling and sampling procedures followed protocols approved by the *Comissão de Ética no Uso de Animais* (CEUA) of the *Instituto de Biociências*, *Universidade de São Paulo* (IB/USP), protocol No. 315/2018. Because *P. vimboides* is classified as “vulnerable” by SALVE/ICMBio [4] and by the IUCN [5], field collections were conducted under authorization from the Brazilian federal environmental agency (ICMBio), responsible for conservation policy implementation and threatened species protection (authorization No. 34938-6).

## 3. Results

### 3.1. Broodstock Selection, Hormonal Induction and Spawning

In the present study, all broodstock subjected to hormonal induction responded positively to artificial reproduction, showing successful spawning or spermiation under the applied protocol. However, survival rate differed between sexes, being relatively low in females (33.33%) compared with males (83.33%). *P. vimboides* exhibited external fertilization and produced spherical, grayish, pelagic eggs.

Spawning by the dry method occurred within 209–230 UTA at approximately 21 °C, with about 1,140,100 eggs released, and the fertilization rate was 93.50 ± 1.32%. Embryonic stages were subsequently analyzed during egg incubation in fiberglass conical incubators maintained at 21.49 ± 0.15 °C, allowing detailed characterization of development under this thermal condition.

### 3.2. Characteristics of the Freshly Spawned Egg and Embryonic and Larval Developmental Stages

From fertilization to hatching, corresponding to the egg stage, embryogenesis followed a sequential progression including zygote, cleavage, morula, blastula, gastrula, organogenesis, and hatching (Table 2). All stages and associated behavioral observations were characterized with precise timing and brief descriptions provided in Table 2. Developmental heterogeneity among embryos was observed, with different stages occurring simultaneously within the same batch. To account for this asynchrony, each stage was defined when 50% of eggs, embryos, or larvae exhibited the corresponding morphological characteristics. Time after fertilization (AF) for each stage is indicated in parentheses.

**Figure 2:**
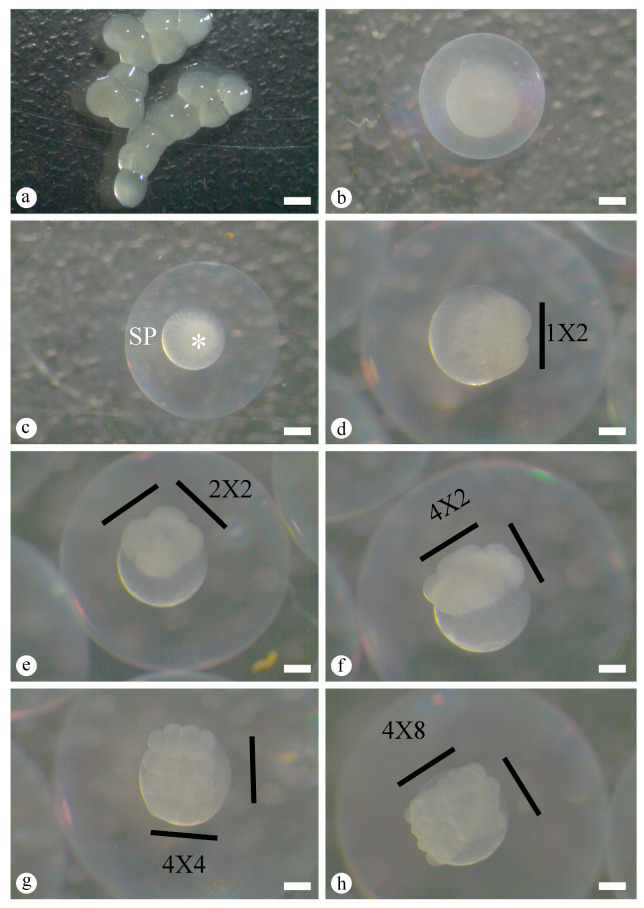
Macroscopic characteristics of embryonic development of *Prochilodus vimboides*. (**a**) The oocyte extruded immediately after spawning, prior to fertilization and hydration (2.58 ± 0.09 mm). (**b**) Hydrated egg shortly after fertilization (AF), time zero AF (4.29 ± 0.14 mm). (**c**) Activated egg showing animal pole (asterisk) and perivitelline space (SP). (**d**) Two-blastomere stage, 20 min AF. (**e**) Four-blastomere stage, 42 min AF. (**f**) Eight-blastomere stage, 1 h 13 min AF. (**g**) Sixteen-blastomere stage, 1 h 36 min AF. (**h**) Thirty-two blastomere stage, 1 h 55 min AF. Scale bars: (**a**) 1.77 mm, (**b**,**c**) 0.88 mm, and (**d**–**h**) 0.55 mm.

During the zygote stage, before hydration, the egg was grayish, non-adhesive, and ovoid, with an average diameter of 2.58 ± 0.09 mm (Figure 2a). After fertilization and hydration, egg diameter increased to 4.29 ± 0.14 mm (Figure 2b), and a large perivitelline space became clearly distinguishable at 15 min AF. At this stage, blastodisc formation was evident as cytoplasmic streaming toward the animal pole, where the nucleus is located, producing a prominent cytoplasmic layer known as the blastodisc. The activated egg therefore exhibited a large transparent perivitelline space (SP), and a distinct animal pole, where embryogenesis was initiated (Figure 2c).

**Figure 3:**
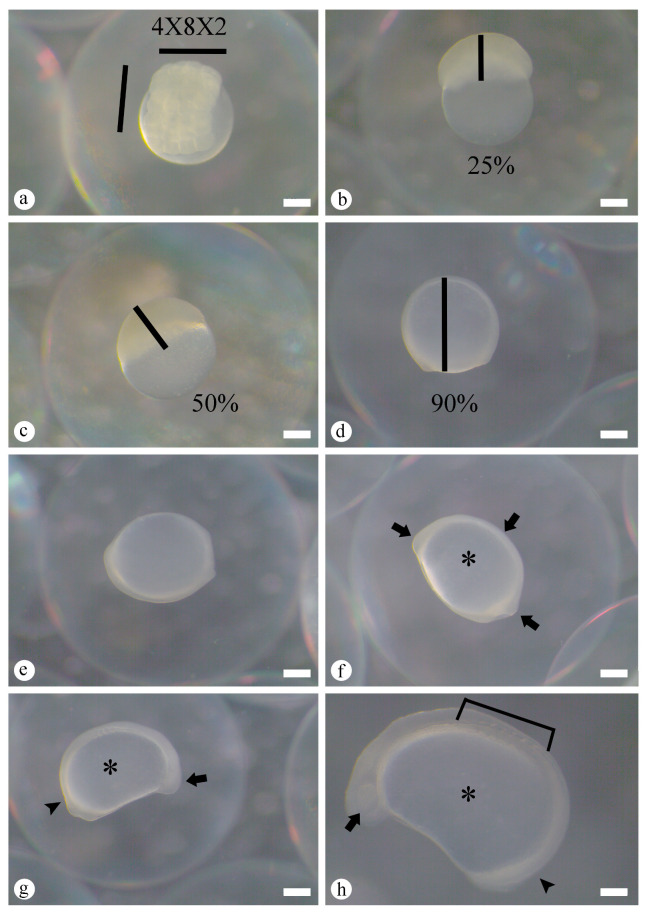
Macroscopic features of embryonic development of *Prochilodus vimboides*. (**a**) Sixty-four blastomere stage, 2 h 14 min after fertilization (AF). (**b**–**d**) Gastrula stage: 25% epiboly (3 h 23 min AF) (**b**), 50% epiboly (6 h 25 min AF) (**c**), and 90% epiboly (9 h 11 min AF) (**d**). (**e**) Final gastrula with blastopore closure, 11 h 47 min AF. (**f**) Embryo differentiation showing embryo (arrow) and yolk sac (asterisk), 12 h 55 min AF. (**g**) Detail of cephalic (arrow), caudal (arrowhead), and yolk sac (asterisk) regions. (**h**) Differentiated cephalic region with optic vesicle (arrow), caudal region (arrowhead), visible somites (delimited area), and yolk sac (asterisk), 19 h 18 min AF. Scale bars: (**a**–**g**) 0.55 mm and (**h**) 0.34 mm.

Cleavage began at 20 min AF and was characterized by formation of a cellular region above the yolk mass. The blastodisc divided symmetrically into two blastomeres (first cleavage, 1 × 2 arrangement, 20 min AF, Figure 2d). Subsequent cleavages produced four blastomeres (second cleavage, 2 × 2 arrangement, 42 min AF, Figure 2e), eight blastomeres (third cleavage, 4 × 2 arrangement, 1 h 13 min AF, Figure 2f), sixteen blastomeres (fourth cleavage, 4 × 4 arrangement, 1 h 36 min AF, Figure 2g), and thirty-two blastomeres (fifth cleavage, 4 × 8 arrangement, 1 h 55 min AF, Figure 2h). The sixth cleavage produced sixty-four blastomeres (4 × 8 × 2 arrangement, 2 h 14 min AF, Figure 3a), at which point individual blastomeres remained distinguishable. Beyond this stage, cleavage became progressively more complex and stereotypical blastomere arrangements were no longer discernible, because some blastomeres overlapped others and cell cycles lost synchrony and spatial organization.

The morula stage was characterized by continued cell divisions beyond sixty-four blastomeres, forming a compact, “half-berry”-like structure. At this point, both the periblast and blastoderm regions were identifiable, representing the transition to the blastula stage. Progression within the blastula stage involved a “cup”-like arrangement of blastomeres and the onset of cellular movements without clear boundaries between embryonic cells. Gastrulation began with epiboly, a morphogenetic movement in which the blastoderm spread over the yolk mass, establishing the germinal layers and embryonic axis. Epiboly progressed through three phases, 25% epiboly at 3 h 23 min AF (Figure 3b), 50% epiboly at 6 h 25 min AF (Figure 3c), and 90% epiboly at 9 h 11 min AF (Figure 3d), culminating in blastopore closure at 11 h 47 min AF (Figure 3e). The morula and blastula phases together extended from 2 h 14 min to 11 h 47 min AF.

**Figure 4:**
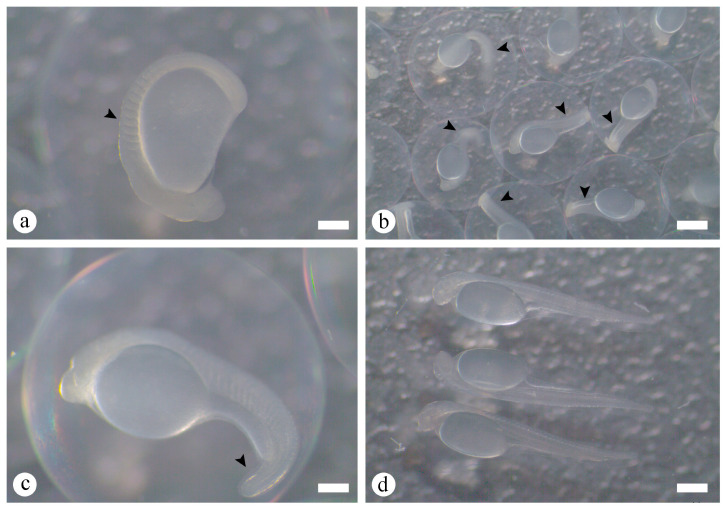
Macroscopic aspects of embryonic development of *Prochilodus vimboides*. (**a**) Detail of somite development (arrowhead) (20 h 04 min). (**b**) Complete segmentation and tail release (arrowhead) (20 h 23 min AF). (**c**) Detail of tail release from the yolk sac (arrowhead). (**d**) Onset of hatching (time zero after hatching—AH) (20 h 34 min) and complete hatching (22 h 04 min). Scale bars: (**a**,**c**) 0.55 mm, (**b**) 1.77 mm, and (**d**) 1.10 mm.

Organogenesis followed gastrulation and was subdivided into early segmentation and late segmentation phases between 12 h 55 min and 20 h 23 min AF (Figure 3f–h and Figure 4a–c). During early segmentation, cephalic and caudal regions formed after establishment of the embryonic axis, and the short tail remained attached to the yolk mass (12 h 55 min to 19 h 18 min AF, Figure 3f,g). Along this axis, non-pigmented regions were visible. At this stage, structures such as the optic vesicle and somites, precursors of axial musculature, became clearly discernible (Figure 3h and Figure 4a). Early segmentation ended when the tail completely detached from the yolk mass and embryo elongation began (Figure 4b, and detail in Figure 4c). These embryos also exhibited muscular contractions (Figure 4b). The late segmentation phase was therefore characterized by a fully extended tail free from the yolk sac, and conspicuous muscular activity preceding hatching.

The hatching stage began at 20 h 34 min AF. Intense muscular contractions contributed to rupture of the chorion, which had become softened and thin. Repeated twitching movements resulted in tearing of the chorionic membrane and release of the larva, representing the onset of hatching (Figure 4d). Complete hatching (100%) occurred at 22 h 04 min AF. Newly hatched larvae measured 5.96 ± 0.06 mm in total length and had a yolk sac volume of 2.876 ± 0.083 mm^3^, initiating the larval stage with yolk sac.

**Figure 5:**
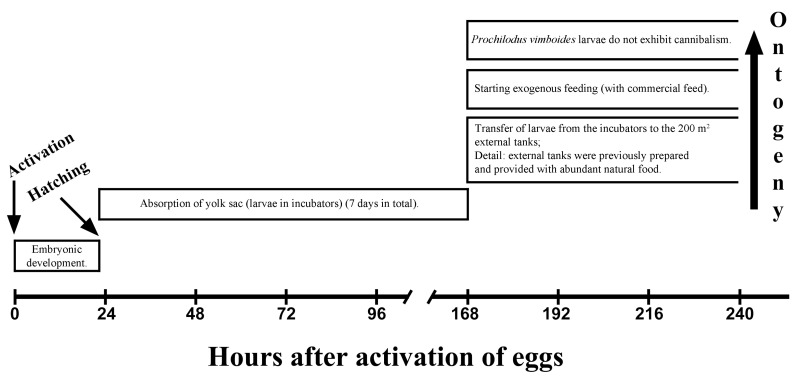
Major ontogenic events during larval development of *Prochilodus vimboides*.

Larvae at hatching had an average length of 5.96 ± 0.06 mm and a yolk sac volume of 2.876 ± 0.083 mm^3^. Immediately after hatching, larvae were translucent and non-pigmented. At the former CESP facility, yolk sac absorption during this phase occurred in fiberglass conical incubators and lasted approximately seven days post-hatching. After this period, larvae were transferred from incubators to outdoor earthen ponds of 200 m^2^ that had been previously prepared to ensure abundant natural food availability. The timing of major events during the early development of *P. vimboides* is summarized in Figure 5.

## 4. Discussion

Despite the environmental relevance of *P. vimboides*, its ecological importance within the Paraíba do Sul River Basin, and its threatened conservation status [6], few studies have used *P. vimboides* as a biological model. For the continuity and long-term success of restocking programs in this basin, greater investment in understanding the morphophysiological traits of early life stages are required. As emphasized in the 1970s by Hempel [28], research on embryonic development, eggs, and larvae of teleost fishes is fundamental for advancing knowledge of basic biology, particularly with respect to ontogenetic variation in morphology, growth, reproduction, feeding, and behavior. In the present study, we provide a detailed description of the embryonic development of *P. vimboides*, one of the five target species of the PAN Paraíba do Sul. To date, only *Steindachneridion parahybae* has been described [11].

Water quality parameters recorded in this study were comparable to those reported for other Neotropical species, particularly within Prochilodontidae [10]. However, the experiments were conducted at a lower water temperature than those reported in previous studies of the genus *Prochilodus* (Supplementary Material), reflecting the relatively low temperatures characteristic of the Paraíba do Sul River [7]. Because teleost fish are ectothermic, water temperature directly influences UTA and embryonic developmental rate, and timing can differ among species [24]. When comparing the temperature and UTA values observed here with those in the literature, it is evident that the temperature regime of the Paraibuna River, which supplies the former CESP fish farm, is lower than that of many other systems. Consequently, UTA and embryogenesis are comparatively slower in *P. vimboides*. In the present study, UTA values for spawning and gamete release, 209–230 UTA at approximately 21 °C, were higher than those reported for *Salminus hilarii* (148.5 ± 13.5 UTA, 26 °C) [29], *Salminus brasiliensis* (150 UTA, 26 °C) [22], *Brycon orbignyanus* (143 UTA, 24.2 °C) [30], and *Brycon cephalus* (182 UTA, 26 °C) [31]. In contrast, *S. parahybae* maintained at the same fish farm exhibited similar UTA values (231 UTA, 24 °C) [11], suggesting that the cooler thermal conditions of the Paraíba do Sul River Basin influence embryogenesis in teleost fishes inhabiting these rivers. From an applied perspective, the temperature-specific embryological timeline reported here, 209–230 UTA at approximately 21 °C, provides a practical framework to optimize incubation temperature, handling schedules, and timing of larval management in captive breeding programs. This can reduce stress and improve hatchery efficiency in *P. vimboides* and other rheophilic species [10,24].

Induced reproduction of *P. vimboides* using cPE followed by the dry method is recommended for embryological studies, because it enables precise determination of fertilization timing and detailed monitoring of developmental progression. However, this approach produced marked differences in post-spawning survival between sexes, with higher survival observed in males (83.33%) than in females (33.33%). This discrepancy may be associated with the greater physiological and energetic demands imposed on females during final oocyte maturation, ovulation, and egg extrusion, as well as stress related to handling and invasive procedures. Similar patterns of higher post-extrusion mortality in females have been reported for other Neotropical species subjected to induced reproduction, including *P. lineatus*, *P. argenteus*, *P. brevis*, *P. costatus*, *P. hartii*, *P. magdalenae*, *P. mariae*, *P. nigricans*, *P. reticulatus*, as well as *Brycon orbignyanus*, *Leporinus macrocephalus*, *Piaractus mesopotamicus*, and *S. brasiliensis* [32,33,34,35,36,37,38,39,40,41,42,43]. In contrast, natural spawning after hormonal induction has been shown to reduce post-spawning mortality by minimizing handling stress [44,45]. Therefore, while the dry method remains appropriate for embryological analyses, natural spawning is recommended for broodstock management and conservation programs, particularly for threatened rheophilic species such as *P. vimboides*.

The earliest stages, from fertilization to zygote formation and early cleavage, are particularly sensitive to environmental conditions, especially temperature. In *P. vimboides*, the zygote and cleavage stages occurred within approximately 2 h AF, similar to that reported for the congeners *P. lineatus* [33] and *P. brevis* [37], and for other Characiformes, such as *S. hilarii* [29] and *S. brasiliensis* [22]. In contrast, Siluriformes such as *S. parahybae* [11] required slightly longer under comparable thermal conditions, approximately 2 h 20 min. The cleavage and blastula phases represent critical periods of embryogenesis, because they involve rapid mitotic activity and establishment of embryonic layers such as the blastoderm and periblast. The periblast, also known as the yolk syncytial layer [46], is essential for yolk mobilization and plays a central role in nutrient absorption during endogenous feeding [47]. In *P. vimboides* in the present study, as well as in *S. hilarii* [29] and *S. parahybae* [11], abnormalities during cleavage and blastula have been associated with increased deformity rates and mortality, reinforcing the importance of stable physical-chemical water conditions during artificial reproduction, as discussed for other teleosts [33,48]. In addition, increased mortality was observed for *P. vimboides* during these developmental stages. These differences underscore species-specific embryonic physiology, because rates of cleavage and epiboly reflect both ectothermic sensitivity to temperature and evolutionary aspects of reproductive strategy [49].

The gastrula phase, defined by epiboly and blastopore closure, represents one of the most physiologically demanding stages and varies across fish species. Cellular rearrangements during gastrulation establish the body axis and germ layer organization [50]. Previous studies commonly classify gastrulation by epiboly percentage, including 25%, 50%, and 90% progression until blastopore closure [11,29,33,51]. In *P. vimboides*, this phase lasted nearly 12 h AF, which is similar to reports for other Prochilodontidae species (Supplementary Material). In contrast, gastrulation in *S. hilarii* (Characidae) [29] occurred from 3 h 56 min to 9 h 01 min AF at 26 °C, in *B. cephalus* (Bryconidae) [31] from 1 h 45 min to 7 h 45 min AF at 26.8 °C, and in *Pimelodus maculatus* (Pimelodidae) [51] from 2 h 15 min to 5 h AF at 29 °C. These comparisons indicate that all cited species exhibited a shorter gastrula phase than *P. vimboides*.

During organogenesis, emergence of the notochord, optic vesicles, and somites establishes the cephalic caudal body axis and allows clear distinction between the embryo and yolk sac [10,50]. In *P. vimboides*, key morphological features were evident during organogenesis, which occurred from 12 h 55 min to 20 h 23 min AF. As in earlier stages, water temperature directly influenced the timing of cephalic and caudal differentiation. Other teleosts exhibit variable timing for this process, including *P. maculatus* with differentiation after 10 h 50 min at 23.1 °C [52], *S. parahybae* after 11 h 20 min at 24 °C [11], *Pseudoplatystoma corruscans* after 7 h 50 min at 23.5–25 °C [53], and *Rhamdia hilarii* after 7 h 30 min at 24 °C [54]. Toward the end of organogenesis in *P. vimboides*, the tail became fully released from the yolk sac, muscular segmentation was apparent, and intense muscular activity contributed to chorion rupture and initiation of hatching. Tail release coupled with muscular contractions initiating hatching has been reported across teleosts, with timing largely influenced by temperature, and lower temperatures generally extending the hatching period [55].

In the present study, complete hatching occurred at 22 h 04 min AF at 21.49 ± 0.15 °C. For the same species, but for individuals from an ex situ bank of endangered species in the Imbé River at Itaocara City, Rio de Janeiro State, Brazil, *P. vimboides* hatched at 24 h AF at 26.5 ± 1.41 °C [13]. Although temperature was higher in that study, larvae required a longer time to hatch. According to Penney et al. [56], hatching time may reflect life-history strategy traits associated with environmental particularities and species-specific developmental rates. The delayed hatching observed in *P. vimboides* may be advantageous, because larvae emerge at a larger size than those of other *Prochilodus* species (Supplementary Material). However, it remains difficult to assess the relative contributions of environmental factors beyond temperature, including photoperiod, pH, and dissolved oxygen. A limitation of the present study is that embryonic development was characterized for a single population under a specific thermal regime, and developmental timing may therefore vary among populations exposed to different environmental conditions across the species distribution range [56].

Newly hatched *P. vimboides* larvae were grayish and non-adhesive, had a relatively medium-sized yolk sac, and lacked pigmentation in the body and eyes. No evidence of cannibalistic behavior was observed during the larval phase, supporting the hypothesis that absence of cannibalism may be a characteristic of the genus *Prochilodus*, in contrast to other teleost genera [10], as reported for *S. hilarii* [29], *S. brasiliensis* [22], *S. parahybae* [11], and *B. cephalus* [31], among others [57]. Post-hatching physiology also differed in important ways. *P. vimboides* larvae hatch with a medium-sized yolk sac that provides endogenous reserves and prolongs the period before the onset of exogenous feeding. This feature is advantageous in *P. vimboides* aquaculture because it allows greater flexibility in feeding schedules. Nonetheless, the transition from endogenous to exogenous feeding remains a critical bottleneck. At the former CESP fish farm, larvae were maintained in incubators for seven days and then transferred to outdoor tanks previously prepared to provide natural food, followed by initiation of exogenous feeding with commercial feed (Figure 5). According to Dabrowski [58], larvae of some species initially lack full gastrointestinal functionality and rely on enzymatic activity associated with live food, a process termed exogenous enzymatic supplementation. Therefore, outdoor tanks prepared to provide abundant natural food are essential for early survival in *P. vimboides* with inert diets introduced gradually.

Overall, these findings provide relevant information to improve larval development protocols, feeding strategies (including the transition from endogenous to exogenous feeding), and rearing practices, and to support species-specific conservation management for this threatened species in the Paraibuna River region, São Paulo State. Ontogeny and embryonic development of *P. vimboides* were largely consistent with patterns described for other *Prochilodus* species. Although different temperature regimes were not experimentally tested in the present study, comparative analysis with published data for other *Prochilodus* species (Supplementary Material) and with *P. vimboides* from a distinct population under a different thermal regime reported by Souza et al. [13,14] suggests that water temperature likely influences the timing of developmental events. In addition, the present findings, together with previous observations by Souza et al. [13,14], provide a foundation to improve reproduction and larval survival in captivity, enabling large-scale production and directly supporting conservation programs in the Paraíba do Sul River Basin. Nevertheless, additional studies addressing basic biology, reproductive physiology, and early stage nutrition remain necessary to further improve production. This study contributes critical biological knowledge for conservation biologists and aquatic physiologists, and supports strategies to predict, prevent, and address future challenges in the recovery of this threatened Neotropical fish.

## 5. Conclusions

In Brazilian aquaculture practice, the embryological and physiological information generated here can be directly applied to improve hatchery protocols. For *P. vimboides*, successful reproduction requires hormonal induction followed by hand-stripping and dry fertilization, which are recommended for embryological studies, as performed in the present study. However, hormonal induction followed by natural spawning is feasible and is recommended for broodstock management and conservation programs. Accordingly, this study characterized the ontogeny and organogenesis of early development in *P. vimboides*.

## Figures and Tables

**Table 1:** Water quality parameters recorded during artificial reproduction of *Prochilodus vimboides* with two intraperitoneal injections of carp pituitary extract (cPE) at the former *Unidade de Hidrobiologia e Aquicultura* of the *Companhia Energética de São Paulo* (CESP) fish farm. Data are presented as mean ± standard error of the mean (M ± SEM).

Period of Artificial Reproduction	Water Temperature (°C)	Dissolved Oxygen (mg L^−1^)	pH	Conductivity (mS cm^−1^)
First injection (cPE kg^−1^)	21.43 ± 0.05	6.84 ± 1.87	6.82 ± 0.28	0.043 ± 0.04
Second injection (cPE kg^−1^)	21.77 ± 0.80	6.58 ± 1.47	6.02 ± 0.67	0.050 ± 0.09
Spawning	21.26 ± 1.15	6.71 ± 0.96	6.79 ± 0.44	0.046 ± 0.04
Embryonic development	21.49 ± 0.15	6.69 ± 0.06	6.64 ± 0.21	0.050 ± 0.02
Hatching	20.76 ± 0.98	6.64 ± 1.49	6.94 ± 0.60	0.046 ± 0.06
First seven days (larvae in incubators)	20.79 ± 0.16	6.69 ± 0.03	6.79 ± 0.06	0.040 ± 0.01

**Table 2:** Developmental timing of the principal morphological events and brief descriptions observed during embryonic and larval development of *Prochilodus vimboides* at the former *Unidade de Hidrobiologia e Aquicultura* of the *Companhia Energética de São Paulo* (CESP) fish farm during the reproductive season.

Time After Fertilization	Stage	Brief Description
00 h 00 min	Zygote	Following fertilization, cytoplasmic streaming toward the animal pole led to the formation of a distinct blastodisc, while a well-defined perivitelline space became evident.
00 h 15 min	Zygote	Characterized by the presence of a large perivitelline space, which persisted throughout early embryonic development at the animal pole.
00 h 20 min	Cleavage	Up to 50% of embryos exhibiting two blastomeres arranged in a 1 × 2 configuration.
00 h 42 min	Cleavage	Up to 50% of embryos presenting four blastomeres organized in a 2 × 2 arrangement.
01 h 13 min	Cleavage	Up to 50% of embryos showing eight blastomeres arranged in a 2 × 4 configuration.
01 h 36 min	Cleavage	Up to 50% of embryos exhibiting 16 blastomeres arranged in a 4 × 4 pattern.
01 h 55 min	Cleavage	Up to 50% of embryos presenting 32 blastomeres organized in a 4 × 8 configuration.
02 h 14 min	Cleavage/morula/ blastula	Up to 50% of embryos exhibiting 64 blastomeres arranged in a 4 × 8 × 2 pattern; the morula stage was defined by the presence of more than 64 blastomeres, whereas the blastula stage was identified by the clear differentiation of the periblast and blastoderm regions, persisting until the onset of gastrulation.
03 h 23 min	Gastrula	Initiation of coordinated cell movements with clearly defined embryonic cell boundaries; up to 50% of embryos exhibiting approximately 25% epiboly, indicating morphogenetic movements that reposition the blastoderm relative to the yolk mass and establish the embryonic axis.
06 h 25 min	Gastrula	Up to 50% of embryos reaching approximately 50% epiboly.
09 h 11 min	Gastrula	Up to 50% of embryos exhibiting approximately 90% epiboly.
11 h 47 min	Gastrula	Blastopore closure observed when the yolk mass became completely enveloped by the blastoderm, resulting in the formation of the yolk sac.
12 h 55 min	Organogenesis	Establishment of the embryonic axis accompanied by a clear differentiation of the yolk sac.
19 h 18 min	Organogenesis	Clear distinction between cephalic and caudal regions, with the tail still attached to the yolk sac; somites clearly evident.
20 h 04 min	Organogenesis	Optic vesicles visible, accompanied by an increased number of somites.
20 h 23 min	Organogenesis	Tail fully detached from the yolk sac; embryo exhibiting pronounced muscular contractions.
20 h 34 min	Hatching (beginning)	Vigorous muscular activity leading to rupture of the chorion and subsequent larval release; conspicuous pigmentation observed in the ventral and encephalic regions.
22 h 04 min	Hatching	100% of larvae hatched, with a total length of 5.96 ± 0.06 mm and a yolk volume of 2.876 ± 0.083 mm^3^.

## Data Availability

The data presented in this study are available in the article.

## Supplementary Materials

The following supporting information can be downloaded at https://www.mdpi.com/article/10.3390/ani16050852/s1: File S1 (EXCEL FILE). References [1,10,13,14,33,34,35,36,37,38,39,40,41,42,43,59,60,61,62,63,64] are cited in the Supplementary Materials.
